# Revisioning Clinical Psychology: Integrating Cultural Psychology into Clinical Research and Practice with Portuguese Immigrants

**DOI:** 10.3389/fpsyg.2013.00164

**Published:** 2013-05-16

**Authors:** Susan James, Sara Harris, Gary Foster, Juanne Clarke, Anne Gadermann, Marie Morrison, Birdie Jane Bezanson

**Affiliations:** ^1^Department of Educational and Counselling Psychology, and Special Education, University of British ColumbiaVancouver, BC, Canada; ^2^Department of Philosophy, Wilfrid Laurier UniversityWaterloo, ON, Canada; ^3^Department of Sociology, Wilfrid Laurier UniversityWaterloo, ON, Canada

**Keywords:** cultural psychotherapy, cultural psychology, culture, psychotherapy, counseling, Portuguese, immigrants

## Abstract

This article outlines a model for conducting psychotherapy with people of diverse cultural backgrounds. The theoretical foundation for the model is based on clinical and cultural psychology. Cultural psychology integrates psychology and anthropology in order to provide a complex understanding of both culture and the individual within his or her cultural context. The model proposed in this article is also based on our clinical experience and mixed-method research with the Portuguese community. The model demonstrates its value with ethnic minority clients by situating the clients within the context of their multi-layered social reality. The individual, familial, socio-cultural, and religio-moral domains are explored in two research projects, revealing the interrelation of these levels/contexts. The article is structured according to these domains. Study 1 is a quantitative study that validates the Agonias Questionnaire in Ontario. The results of this study are used to illustrate the individual domain of our proposed model. Study 2 is an ethnography conducted in the Azorean Islands, and the results of this study are integrated to illustrate the other three levels of the model, namely family, socio-cultural, and the religio-moral levels.

## Introduction

Culture is given increased attention in psychology (Cole, 1996; Leong and Lopez, 2006; Sue and Sue, 2007) as a result of increasing diversity, immigration, and globalization. The American Psychiatric Association (APA) (2000) and Canadian Psychological Association (CPA) (2000) both stress the need for cultural competency in their ethics codes. American Psychological Association (APA) (2002, 2003) and Canadian Psychological Association (CPA) (2001) have adopted guidelines for multicultural practice, and counseling psychologists have identified specific multicultural competencies (Sue et al., 1998). Addressing cultural components of the client-therapist relationship is key to increasing the effectiveness of therapy (Comas-Diaz, 2006). Examples to date of how that has been done include Seeley’s (2000) model of cultural psychotherapy, which adopts an ethnographic approach to cultural issues in therapy.

One way to effectively address culture in therapy is to develop a research-driven comprehensive model with ecological validity that can be applied in a practical setting. We used cultural psychology as the theoretical framework on which to build a program of research with Portuguese immigrants. Based on the results of our research, we developed a comprehensive cultural psychotherapeutic model. Examples from our research with Portuguese immigrants will illustrate aspects of this model.

One-fifth of Canada’s population consists of immigrants; approximately a quarter of a million people immigrate to Canada every year (Statistics Canada, 2008). While research on culturally competent mental health-care is burgeoning, it lags behind the needs of our increasingly diverse population. A significantly sized culture group within that population is Portuguese Canadians, with significant waves of immigration in the 1950s and 1960s. This immigration pattern has formed unique Portuguese communities where language and customs are preserved and where second and third-generation Portuguese Canadians negotiate two cultures. As members of this group tend to be reluctant to seek help outside of the family, their mental health needs often go unmet (Morrison and James, 2009). This ethnic group also has a unique mental health profile, which includes syndromes not found in any other culture and not described by North American psychiatric diagnostic criteria (James, 2002).

Cultural psychology integrates psychology and anthropology in order to enable a complex understanding of both culture and the individual within his or her cultural context. Cultural psychology examines the ways in which “cultural traditions and social practices regulate, express, and transform the human psyche” (Shweder, 1991, p. 73). The notion of intentionality is central to cultural psychology (Shweder, 1991). Intentionality suggests that our experience is never independent of the presuppositions we bring to that experience. Instead, to acknowledge our presuppositions is to acknowledge that we intentionally engage with and create our reality. The socio-cultural milieu becomes the intentional world that humans have created to define their experience and from which they draw meaning and resources. The psyche represents the intentional person, which is unique – global presuppositions cannot be drawn.

In contrast to the concept of intentionality put forward by cultural psychology, traditional mainstream psychology presumes that there are universal laws of nature. Universalized assumptions relevant to psychotherapy and counseling are that (a) the only agency is human agency, (b) language use is epiphenomenal, and (c) western theories of diagnostic classification and healing systems apply globally (Shweder, 1991).

**Figure 1:**
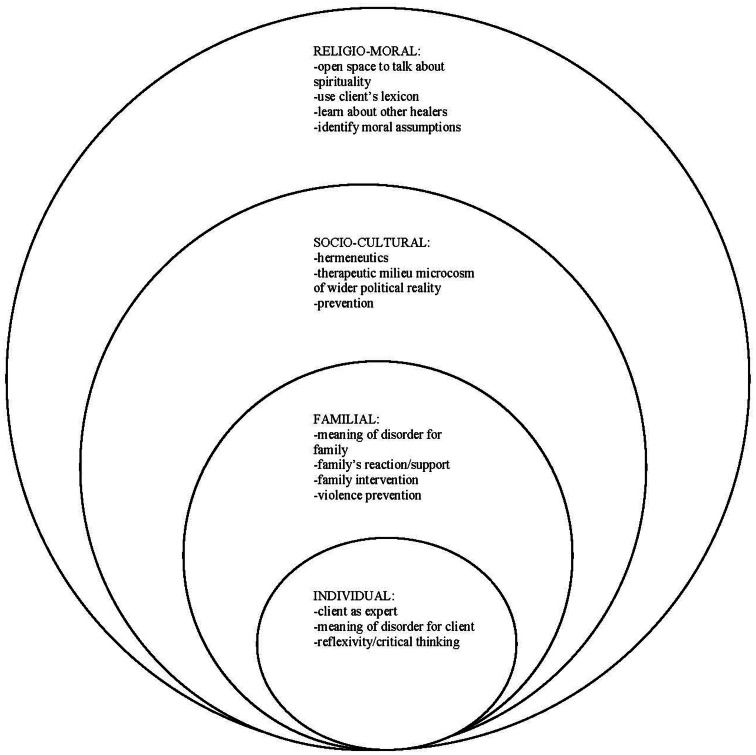
**A multidimensional model of cultural psychotherapy and counseling with Portuguese immigrants**.

Our research with Portuguese immigrants investigated these assumptions. Based on the outcomes of our research, we propose a model for clinical work with Portuguese immigrants. Our model (as depicted in Figure 1) focuses on understanding the person in a multicontextual way. This is reminiscent of Bronfenbrenner’s (1979) concept of an individual’s ecological environment that consists of a complex configuration of nested structures. Szapocznik and Kurtines (1993, p. 400) support this with the notion that the individual is “embedded within a family that is itself embedded in a culturally diverse context.”

Our model utilizes these four contexts (individual, familial, socio-cultural, and religio-moral). We will explore each level with the understanding that all levels are interrelated and significant to the individual.

## Study 1: The Individual Domain

As discussed earlier, there is an intentionality of both the individual and her or his changing world (Shweder, 1991). The individual is an active participant in this changing world, acknowledging, constructing, and reconstructing her or his mentally constituted reality. This reality is a unique interaction of the individual’s previous experience and the culture of the surrounding world. Each individual’s reality is the product of the ways in which she or he contributes to, interprets, and engages the world.

Our work with Portuguese immigrants in North America revealed that traditional psychiatric categories are limited when trying to classify Portuguese idioms of distress (James et al., 2009) such as agonias (James and Clarke, 2001; James, 2002; James and Prilleltensky, 2002; James et al., 2006). Our research into agonias included Portuguese men and women who came from the Azorean Islands and who are now living in the United States (James, 2002) and Canada (James et al., 2005). We explored their understanding of agonias using ethnographic interviews and quantitative methods. James (2002) first demonstrated that agonias is undocumented but very common among Portuguese immigrants. The lack of research directly affects the work of clinicians, as the discrepancy between lay and professional understandings of agonias exacerbates potential misdiagnoses, non-compliance and, consequently, ineffective treatment. For example, many community members stated that agonias was faltando de ar, or missing air. Vision impairment, “burning from within,” insomnia, and an inability to eat were additional symptoms reported by the community. Agonias was attributed to a range of causes, from religious beliefs (as God- given), the social milieu (e.g., spousal mistreatment), to the physical domain (e.g., bad heart, liver problems). Community members discussed cures in terms of teas and or communion with God (prayer) or with their peers. This suggests that agonias cannot be packaged in terms of the North American Diagnostic Statistic Manual (DSM-IV-TR) because it expands beyond the psychological domain to include a myriad of relevant cultural considerations.

In order to provide health practitioners with a tool to assess agonias, the “Agonias Scale” was developed (James et al., 2004). Items were formulated based on the aforementioned ethnographic interviews. The Agonias Scale consists of 13 items, which correspond with symptoms of agonias (see Table 1). Respondents rate the severity with which they experienced symptoms of agonias during the past week on a 4-point scale ranging from “not at all” (0) to “severely” (3) (i.e., the total scale score can range from 0 to 39). In a quantitative study (James et al., 2004), these items were validated with a sample of 201 Portuguese immigrant women living in the Waterloo Region, ON, Canada. In this section of the article, we present data that validates the Agonias Scale with Portuguese immigrant men. Specifically, we investigated the reliability and factor structure of the Agonias Scale as well as how it relates to measures of depression and anxiety in a sample of Portuguese immigrant men. In line with the results for the women, we hypothesized the Agonias Scale to be unidimensional. In addition, we expected the Agonias Scale to show positive correlations to measures of anxiety and depression, as all three are idioms of distress. We hypothesized that the correlations would be of small or medium effect size as previous qualitative research with community members has shown that agonias is distinct from the symptoms of depression and anxiety (James and Prilleltensky, 2002).

**Table 1:** **Factor loadings of the items of the Agonias Scale**.

Item	Factor 1
1. Bad nerves	0.68
2. Heart pain	0.66
3. Chest pain	0.79
4. Difficulty breathing	0.80
5. Premonition that something bad was going to happen	0.71
6. Feeling afflicted	0.88
7. Nausea	0.70
8. Burning from within	0.80
9. Difficulty seeing	0.75
10. Fainting	0.82
11. Difficulty eating	0.66
12. Hot flashes	0.82
13. Fever	0.50

### Method

The sample consisted of 151 Portuguese immigrant men living in the Waterloo region. Participants were recruited through advertisements in Portuguese stores, community centers, and churches. Inclusion criteria for participation were a minimum age of 18 years, Portuguese speaking, and being first generation Portuguese immigrant (i.e., born in Portugal). The mean age of the 148 participants who reported their age was 44.3 years (with a standard deviation of 16.3). The mean years of living in Canada of the 147 participants that responded to this item was 24.7 (with a standard deviation of 12). Participants had the option of responding either in Portuguese or English; the majority of the participants chose to respond in Portuguese.

#### Measures

Besides the Agonias Scale, the following commonly used instruments to assess mental health symptoms were administered to examine the relationship between them and the Agonias Scale.

##### State-trait anxiety inventory

The State-trait anxiety inventory (STAI) (Spielberger et al., 1970) consists of 40-items with a 4-point response scale, with separate subscales of state and trait anxiety (20 items each). For this study, only the state anxiety subscale was used. As the data were ordinal, we computed ordinal coefficient alpha (Zumbo et al., 2007; Gadermann et al., 2012), which was 0.95.

##### Beck depression inventory-IA

The Beck depression inventory (BDI-IA) (Beck and Steer, 1993) is comprised of 21 items that assess depressive symptoms. Each item consists of four statements that are ordered in increasing severity of the depressive symptom. In the present study, ordinal coefficient alpha was 0.91. Both the STAI and BDI-IA have been validated for Portuguese communities by McIntyre et al. (1999).

### Results

Fifty-two percent of the participants reported having experienced some level of agonias during the past week. The most frequently reported symptoms of agonias were bad nerves, difficulty seeing, difficulty breathing, a premonition that something bad was going to happen, and burning from within. The mean score on the Agonias Scale was 2.3 (with a standard deviation of 3.6) ranging from 0 to 18, with a skewness of 2.2 (standard error of 0.2) and kurtosis of 5.0 (standard error of 3.9).

We also examined the factor structure and internal consistency of the measure in this sample. In order to aid the determination of the number of factors, a principal components analysis was conducted. As the data are considered ordinal, the polychoric correlation matrix was used in the software program LISREL. The first eigenvalue was 7.57, explaining 58.23% of the variance; the second eigenvalue was 1.42, explaining 10.93% of the variance, and indicating one dominant component. A parallel analysis indicated that the second random eigenvalue was 1.52, which indicates that the scale was unidimensional. We ran a factor analysis using MINRES on the polychoric correlation matrix for the factor loadings, which are provided in Table 1.

As the factor analysis suggested the Agonias Scale to be unidimensional, we computed ordinal coefficient alpha as estimate of the internal consistency (Zumbo et al., 2007; Gadermann et al., 2012) for the scale, which was 0.94. The correlations between the Agonias Scale and the measures of depression and anxiety were positive and statistically significant. According to Cohen’s (1992) rule of thumb, the correlation between agonias and depression (*r* = 0.39; *p* < 0.01) was of medium effect size, and the one between agonias and anxiety (*r* = 0.26; *p* < 0.01) of small (but close to medium) effect size.

There are a number of implications of these findings at the individual level. Foremost, the client should be treated as the expert. This means that the therapist needs to bracket her or his preconceived notions about the client, the client’s culture, and the client’s experience. The client needs to have space to talk about her or his affective and somatic feelings as she or he experiences them rather than imposing psychiatric categories or emotion terms from western cultural contexts (e.g., anxiety, guilt, fear, envy, love, sadness; Shweder, 2001). Measures such as the Agonias Scale, which are specifically developed to assess symptoms of distress in a culturally sensitive way, are helpful tools to explore the individual and unique experiences of the client through in-depth dialog. Validation of the client’s experience is of special importance in cross-cultural contexts. Clients who feel they are not understood by health-care providers tend to feel confused, disoriented, and may prematurely terminate counseling.

Counselors working in a cross-cultural context should attend not only to clients’ descriptions of their condition, but also to their non-verbal expressions, which are often culturally based and contribute to an understanding of the cultural context and meaning of the client’s experience (Westwood and Ishiyama, 1990). Somatic and affective experiences are rich sources of information for psychotherapists because they are written on the body and indicative of the ethnopsychology of the client. Also, we have found that when we encouraged clients to discuss feelings and idioms, their discussion about healing became more inclusive. For example, when clients talk about agonias, they also often discuss the traditional healers who give them vitamins or prayers to recite (e.g., Nossa Senhora dos Agonia, translated, Our Lady of Sorrows prayer) to alleviate the symptoms of agonias.

This study illustrates the importance of using a mixed-method approach to examine idioms of distress in a culturally sensitive way. Ongoing quantitative and qualitative research is currently being conducted to explore the construction, symptom expression, and treatment of agonias in Portugal.

## Study 2: The Familial, Socio-Cultural, and Religio-Moral Domains

### Study 2a: The family domain

Krause (1995) suggests that family intervention can be useful if therapeutically appropriate and ethical. Krause discusses the notions of generative and interactive aspects of the individual. The former relates to the traditions and histories that are passed down within a specific cultural context and the latter is centered on how the individual reinterprets these traditions based on his or her present reality. Although the generative aspects in a family will be similar, the interactive aspects will vary from person to person. For instance, the family may have a history of Catholicism; however, the ways in which it is manifested and experienced in family members’ daily lives can be idiosyncratic.

For many first generation Portuguese immigrant women, being Catholic involves going to mass at least once a week and praying daily. Children, the second generation immigrants, are often not equally devout in their practice, which can lead to angst in mothers. For example, in James’ (2002) study on agonias, a priest related the following:

Children can give people agonias because they are not living in faith or they are doing things that are forbidden like divorcing, and for the people it’s certainly a weight on them and it gives them a tightening feeling like claustrophobia … Agonias is really anxiety about sin. (James, 2002, p. 96)

This has a tremendous effect on the women and women often tell their families about their suffering. Our previous research with Portuguese Canadians and Portuguese in the Azores (Morrison and James, 2009) also demonstrated the impact of acculturation on generative and interactive cultural practices. Through this research, a model of acculturation was developed that demonstrates how the move to Canada, the passage of time, and generational changes all affect how culture is lived and negotiated in the family. Examples include a generation gap, which develops when children forget or do not learn Portuguese while parents or grandparents are unable to speak English, an increase in family nuclearity, and less openness with regards to discussing family problems with outsiders – including researchers and psychologists.

This nuclearity also plays out at the cultural level and has implications for seeking and receiving help outside of the Portuguese community; informants reported being discouraged by other members of the Portuguese community for doing so. Across all of our research in Canada we found the same patterns of a reluctance of the first wave of immigrants to engage beyond the Portuguese community, and as a result to rely more heavily on the family unit and the ability of the second generation to perform a bridging function when necessary, for example to attend specialist doctors’ appointments. As a result, many individuals of the first wave have lived in Canada for 30 years or more and cannot converse in the English language.

Our previous research had indicated the significant role traditional healers play in the help seeking behaviors of this cultural group. In this study, we sought to understand how traditional healers provided care to families and individuals in emotional distress and how traditional healers conceptualized culture specific disorders such as agonias.

#### Method

Borrowing theories of understanding culture knowledge from Anthropology, we undertook an ethnographic study in the Azorean Islands of Portugal, specifically on the islands of Sao Miguel and Terceira. The participants in this study were people who had been born and raised in the Azores, and who utilized the services of a traditional healer or who had knowledge of traditional healers in the Azores. Nurses, psychologists, physicians, and a professional health-care giver identified as a curandeiro (traditional healer) were also included in the study. On the islands, two local research assistants and one volunteer supported the project by providing access to the community, carrying out formal interviews, and transcribing and translating interview transcripts. Typical of ethnography, a complete demographic list of those who participated in supplying data is difficult. Some informants participated regularly and continued to be a part of the project until the final text was completed. Others were met with once or were simply part of informal conversations. In general, participants ranged in age from early 20s to 50s. In total, 25 formal interviews were conducted with 11 community members and 14 health-care providers. Another aspect of ethnographic research is engagement in community activities. Ervanarias – stores that sell natural products to promote both a healthy body and a healthy mind or bem estar, to be well – were sources of information and shopkeepers were important contributors to our understanding of the psychological health of the community and the people who act as healers. Pamphlets, advertisements, photographs, programs, guidebooks, and artifacts also contributed to our understanding of the context and the process of healing.

#### Results

Our research indicated that in the Azores, suffering brings family and friends together by engaging the community to provide support to the entire family unit. Often it is a family member, a mother or grandmother, who would suggest accessing traditional healers – often after an unsuccessful attempt with a conventional health-care provider. Families often did not feel a physician’s or mental health clinician’s assessment of a situation of distress was accurate or helpful, and often this assessment failed to validate the whole family’s suffering. Notably, reasons to seek help from a traditional healer could also be to address negative family dynamics. Generally, illness was dealt with from within the family unit. One participant described how she and her mother decided to go to a curandeiro for their physical problems:

I had spots like dirt on my neck, greyish and whitish, and other people had that too. Plus my mother also has [had] a problem that she has [had] like pimples on her cheek, once in a while. So we had known this guy was there for many years, but we never, well we heard that other people would go there and because my mother went to so many doctors, medicine for that, for her problem and it didn’t go away. And she decided to go there because some family insisted, “try, try those teas” and because she went, I went with her too.

Quite often, treatment from a traditional healer would involve attendance by all immediate family members. For example, a young boy with symptoms of sleep disruption and severe emotional dysregulation was assessed by a physician as “not sick;” however, he and his family were suffering. When the family sought help from a traditional healer, the healer validated their suffering and engaged each member in the process of healing. The healer remained focused on the discomfort of the family unit and provided care for all those involved.

Another participant described her younger brother’s ailment as one that could not be compartmentalized neatly into one type of illness:

He would not sleep. He would wake up every three hours to have milk, but even after that he would cry all night, unless you would be holding him and going around and around, cause if you sit he would start screaming all over again … Every night at about midnight he would wake up and start pointing at things and screaming.

In this case, a curandeiro told the family that the boy had “the ability to gain powers to [for] good or bad.” He was suffering from distress on a spiritual level, which was causing him physical distress. As well, the young boy’s suffering impacted the whole family. The participant described the boy’s mother as physically exhausted and distraught over her son’s behavior.

Family conflict was also a common reason for seeking the curandeiro’s assistance. One informant described the reasons her friends have gone to curandeiros: One went because she was between two brothers who didn’t like each other, who fight a lot. Another went because her life wasn’t going right. One went because she was pregnant and the families told her that her baby would be born with a physical disability.

Açoreans believe that curandeiros’ ability to heal is a manifestation of the Divine on earth. A health-care professional described this: “I think this [ability] comes through tradition, family to family, many, many, years. He had [has] a connection … between God and the people in this world.” For most, there was no need to understand why a curandeiro has this connection or to prove this connection.

Family therapy can explicate and normalize the intergenerational disagreement. Letting the family know that other families struggle with this challenge can be comforting. It can also be helpful for the family to understand that the same sorts of struggles exist in families in the homeland – especially when parents idealize what life is like in the Azores. The reality is that life has changed there since they left. Bicultural effectiveness training (Szapocznik, 1984) can be useful in this situation. Szapocznik noticed in working with Cuban American immigrants that many problems in families occur because family members acculturate at different rates (for instance, children ahead of parents and boys before girls). Thus, the therapist orchestrates the sharing of each person’s values so that family members can learn about the assumptions of other family members. In this case, this would involve asking the mother about her expectations and the values she is trying to impart as well as understanding the context from which those values came. The other family members can also talk about these values and how they are lived out in North America. The values can be a common ground for dialog between generations because often times they are shared.

The same sort of dialog can occur with regard to bodily symptoms. The therapist can help orchestrate a dialog between family members about each member’s conceptualization of the illness or phenomenon that they are facing. As was stated earlier, there are both cultural traditions and individualistic interpretations of meaning. For example, in the Portuguese community there is an intergenerational disparity in the way illnesses are articulated and treated. Many of the older generation use the folk idiom of distress, agonias. In North America, to be validated by younger family members, illness often has to be framed through a western allopathic lens; support and care ensue if there is a medical diagnosis. Older Portuguese immigrants may seek out indigenous healers, but these are often discredited by younger family members. Family therapists can help the family talk about the varied meanings they give the concept of illness. It is important for the therapist to model the ability to tolerate a diversity of meanings, including those that differ from her or his understanding of illness. Additionally, allowing clients, particularly older generations, to use folk idioms helps validate the understanding of illness that are at the root of these idioms in the eyes of the young.

### Study 2b: The socio-cultural domain

The cultural psychology framework (Shweder, 1991) of intentional persons and intentional worlds reveals that there is interplay between the individual and his world that is consistently evolving, each a function of the other. Specifically, psyche refers to the intentional person and culture refers to the intentional world. Thus, whatever the individual frames in her or his intentional world (for example, culture, social milieu, religious tradition) can become a catalyst for individual transformation and growth. The intentional world is fluid and based on the individual’s interpretation and incorporation of multiple realms. The individual does not exist without a culture. How she or he frames culture, society, and religion determines the ways in which transformation can occur (Shweder and Much, 1987; Markus and Herzog, 1995; Markus et al., 1997; Markus and Kitayama, 1998).

In an attempt to understand how therapists understand their clients’ cultural world, we conducted a study on this topic (Harris, 2001). We explored whether Portuguese mental health providers (*N* = 8) were using a hermeneutic approach to therapy with the Portuguese immigrants. From this study, various themes emerged that align with a hermeneutic process for therapy. The therapists related multiple areas where the Portuguese client was understood in terms of his social and personal milieu. For example, many Portuguese clients described malaise in terms of somatic or concrete symptoms. The Portuguese therapist explained, “It’s hard for them to express their needs and how they’re feeling and why they’re doing such and such. So the way to do that, they have to say I’ve got a headache or this is hurting me” (Harris, 2001). Because discourse on emotions was not generally accepted, Portuguese clients engaged the physical to communicate the emotional. The therapist responded by prescribing wellness in concrete terms. For example, she had a client “put on her best clothes and go out in public” to mediate the client’s feelings of sadness.

Further, therapists’ reactions to cultural idioms of intervention such as traditional healers were also explored. One Portuguese therapist related (as cited in Harris, 2001),

If it makes my patient feel better, I have no reason to argue, you know. I think that being a health provider, my ultimate goal is for the patient to feel better, and it would be nice for me to think that I’m the major source of that wellness, but the patient can also get help from someplace else, by all means. Even though I cannot explain it, even though I might not totally understand it or agree with it.

#### Methods

Study 2b used data collected as part of the same ethnography described in the methods Section “Study 2a: The Family Domain.”

#### Results

The Azorean ethnography revealed a number of new insights at the socio-cultural level. First, the study elucidated the difficulties in attempting to translate the language and concepts of distress from one language to another and from one culture to another. This, what we called the meaning gap, was present on many different levels; medical language to colloquial language, Portuguese language to English language, and medical culture to community culture, Portuguese culture to North American culture. Conventional health-care professionals implied that Azoreans lacked the medical language to express their distress adequately and, therefore, were difficult to treat appropriately. As a researcher and an outsider, I (Bezanson) had difficulty understanding the meaning of the way in which symptoms, causes, and cures were expressed, and I often felt like I was missing something. One physician described another problem: “For some, the words healers used were an important part of healing and if the words were not right, healing would not happen.”

Second, we learned that the curandeiros are well integrated into the community. The curandeiro works with community members to provide healing on three levels: psychologically, spiritually, and physically. This informant described the role of a curandeiro in the Açorean society:

Now, curandeiros have a social role. In the first place they are people from the freguesia [small parishes] … And they have many times, a tranquilizing role because as they [curandeiros] even know the [client] and they know how they live and they know that you have a nervous disease … he is a peer among them. He is a person who has certain knowledge, who developed certain knowledge and sometimes they even bring a certain peace of mind, a certain tranquility. The curandeiro has a little bit the role that the psychologist has in big institutions.

Good curandeiros never ask for money but accept what people give. Referring to a curandeiro, one participant said: “He’s a good person, a great person. He doesn’t take our money. We give what we want.” This participant recalled a situation when she was an adolescent and a curandeiro was called to remove evil spirits from her home, “That is the thing, I don’t know. One thing I know, we never give the money in hand and there is never a price. I don’t know how they agree on the price. It is strange.” No one was able to give me an example of how much services would cost.

Curandeiros were often called on at odd hours and are expected to always be available. This informant is clear about the availability of her curandeiro, “He came at one o’clock in the morning. He goes [comes] anytime we need him.” Another participant, a health-care professional, compared the accessibility of conventional health-care providers with that of a curandeiro:

Most of the time doctors are doctors because of the money. He [curandeiro] doesn’t become a doctor because he has the vocation to be a doctor, help people and save lives. Curandeiros dedicate themselves to the people … sometimes three, four o’clock in the morning, you go knock on these peoples’ [the curandeiros’] door.

Healers in the Azores were members of the community and part of the everyday lives of the people they tried to help.

In the Guidelines on Multicultural Education, Training, Research, Practice, and Organizational Change for Psychologists, the American Psychological Association has recognized that both the client and the therapist are “cultural beings” and that individual attitudes and beliefs are intertwined with the socio-cultural environment [American Psychological Association (APA), 2002, p. 17]. A hermeneutic approach to psychotherapy and counseling situates the client within the context of their multi-layered social reality. Specifically, Guideline #5 suggests a process of addressing the interplay between the individual and her or his world and Guideline #1 expands on this idea by including the intentional world of the therapist in the dynamic of the therapeutic relationship.

In previous articles we described this theory (James and Foster, 2003), research (Harris, 2001), and psychotherapeutic approach (James and Foster, 2003, 2004, 2006; Foster and James, 2004; Christopher et al., 2007) utilizing hermeneutics more fully. A hermeneutic paradigm suggests that the client possesses both personal and cultural histories. Thus, it would be limiting in therapy to make broad generalizations based on the client’s culture of origin. Significant understanding takes place at the intersection of the dialectic between individual articulations of experience (the part) and the broader cultural context of the client (the whole). This notion rests on the assumption that to be human is to possess both an individual as well as a social and cultural identity. From this framework, the therapist determines the most suitable wellness paradigm for each client without making generalizations based on culture, gender, or age group (James et al., 2005).

A therapist must consider the wider political reality of the Portuguese. The Azores has a history of dictatorship that allowed for no overt opposition (James, 2002). In light of this oppression, the resistance of Azorean immigrants in North America to speak up for themselves in the workplace or in therapy is understandable. In therapy, Azoreans tend to respond to questions of their well-being by saying “Voce que sabe,” meaning you are the one who knows. The therapists, trained in North America, often described such clients as “passive” or “passive-aggressive.” Similar patterns in therapy have been observed for African Americans and passiveness (Sue and Sue, 2007), and Native Americans and distrust of counselors (Baruth and Manning, 1999), both having roots in the history of relations with European Americans. Thus, it is important to explore the client’s behavior in relation to their cultural history. It is also important that the therapist employs a culturally reflexive stance, recognizes the inherent limitations of his or her multicultural awareness, and learns ways in which these limitations can be ameliorated (Qureshi, 1999).

### Study 2c: The religio-moral domain

The religio-moral domain is part of the intentional world of the individual. Yet spirituality as a component of psychotherapy has not yet been fully embraced by practitioners. The religious reality of clients cannot be separated from their individual, familial, and social reality; all are interconnected and interdependent.

Although an assumption of many psychotherapy theories is that the only agency is human agency, this was not consistent with our research with Portuguese immigrants (James, 2002; Foster and James, 2004; Bezanson and James, 2009). For many participants, their Judeo-Christian tradition affected all aspects of their lived experience. For instance, James and Prilleltensky (2002) found that community members’ “causal ontology” regarding their suffering was interwoven in the fabric of their religious traditions. Members of the Portuguese immigrant community stated that illness was predicated on their own sin or the sins of family members.

Many informants in our research suggested that just like martyrs, all community members suffer to some degree because the suffering is redemptive, appropriate, and even necessary based on their religious beliefs (James, 2002; Bezanson, 2008). When Portuguese suffer, their family and community suffer with them. However, psychological distress and mental illness are not as visual as diseases of the physical body. This poses a problem in communicating suffering to the world and thus, ensuring that goodness as a Christian is made public. Suffering needs to be witnessed which, on the religio-moral level, positions the community member as a good person in society.

#### Methods

Study 2b used data collected as part of the same ethnography described in the methods Section “Study 2a: The Family Domain.”

#### Results

The research identified five different types of healer in the community; herbalistas (herbalist), endireitas (bonesetter), as mulhers quem ler do livro (the woman who reads the book), bruxas (witch or medium), and curandeiros (healer). Further, the findings suggested the community members felt that conventional health-care systems (which includes mental health clinicians) were not caring for an integral part of their illness, what one informant called “psychological support.” Another informant suggested that by accessing traditional healers, community members were “trying to get what they can’t in medicine.” There was a strong sense that traditional healers provided a more holistic type of care by validating suffering (a necessary component of being a good person, as discussed above) and by providing a physical treatment (herbs, teas, a prescribed action, or massage). Suffering was not enough for the Azoreans; the suffering must somehow be communicated. Traditional healers seemed to provide the necessary conduit to express suffering from within the individual to the outside world. One informant said:

Sometimes I say I don’t know what God has to do with this but the faith comes along … In your case [counseling psychology] and in psychiatry, you need proof to see. New doctors today need proof. And sometimes, this is what I believe. I think if God doesn’t want you to have the truth, you are not going to get it. You can look. I think you can go looking for anything around you but you’re not going to have proof. Because some things can’t be shown to you. Doctors have been stubborn, looking for solutions for this and this and that. And sometimes people have, for example curandeiros, have been giving solutions to people that their own doctors don’t believe in. That is why people most of the time people prefer curandeiros or a naturopathic doctor because most of the time curandeiros and naturopathic doctors agree. Sometimes he closes the eyes … [to] what he learned … and goes to the other science.

The “other science” is considered to be the gift or an ability to know what is wrong and how to fix it. One informant relayed “I think it is the [curandeiro’s] ability of seeing within yourself [the client], if you have envy or all those other things upon you, not [you] having them but other people sending them to you, energies, bad energies.” And they have the ability to see that.

A mental health-care professional explained, “He looks at people and I guess he sees through, he sees inside us and he sees what we have.” Many informants stated that curandeiros are able to look at you and immediately know what is keeping you from good health. One health-care professional believed that the curandeiro’s ability to give him information about his life was proof of the gift:

An Açorean proverb (Emilio, 1990) highlights this trust in God, Deus é quem cura e o médico leva o dineiro [It is God who cures and the doctor who gets the money]. In his well known book Namora (1956), Retalhos da Vida de um Medico [Remnants of the Life of a Doctor; translated version, Mountain Doctor], chronicles his life serving a rural population on mainland Portugal. He wrote of curandeiros, " The doctor was, so to speak, merely a professional without a real vocation; it was safer to trust someone born with the gift. (p. 118) Curandeiros have an ability to understand distress and execute an intervention without gathering information through questioning or utilizing medical or psychological tests. This ability is often called a gift or condão which translates as a special virtue, magic powers, prerogative, mental power, or ability. Most often, the gift is used to know or diagnose the root of distress that a person is suffering simply by looking at them. As one participant mentioned previously, the condão is the ability to see what others cannot see.

The Azorean informants identified the importance and implications of having the gift. Faith is very important for the Portuguese – they rely on God for healing. For the Portuguese community, the goal is not to remove suffering immediately without understanding its web of significance. Through religion, the client can find meaning in her or his suffering. Clients’ lived realities are part of a larger story, creating a context whereby they are validated. To this end, Portuguese community members engaged in storytelling as the principal means for relating their experiences. Discussions about their lived reality (plans, purpose, mores, and suffering) provided a narrative that revealed substantive aspects of their lives (James et al., 2005) and validated their position in their community on a religio-moral level.

Through trial and error, the clinicians involved in our research learned that in contrast to the culture of psychotherapy, suffering has deep spiritual meaning for the Portuguese. For instance, our informants persistently discussed their physical ailments as religio-moral manifestations of being a good person. To them, suffering increases one’s spiritual proximity to God and others. This view provides a challenge for therapists whose attempts to explore exceptions to suffering or set goals to remove suffering will be unwelcomed, which could be conceptualized as resistance or a lack of readiness for change by the therapist. For instance, when I (James) tried to use solution focused therapy with a Portuguese woman who had many physical ailments, her suffering became more intense over the next few sessions instead of less. Then, while interviewing the Portuguese General Practitioners for the research I learned their approach. They realize the meaning attributed to suffering so they do not say that they will relieve all of their suffering. Rather they say “we know that you have diabetes, arthritis, and asthma. Right now we will just work on your symptoms of diabetes to make them a little better.” Once I understood that approach I was able to help the Portuguese woman in therapy.

Other researchers have identified similar religious significance within other cultural groups (West, 2000). Incorporating a narrative therapy approach (White and Epston, 1990) in psychotherapy would address the religio-moral domain of the client and thus provide more sensitive treatment. One form of narrative therapy that is particularly beneficial with this community is “meaning-centered” narrative therapy (Wong, 1989), which not only investigates the client’s story, but also looks at how the story is situated within larger meaningful stories, such as religion. Another reason why narrative therapy is helpful is because telling stories is part of the culture. Talking about feelings, on the other hand, is not part of the lexicon so client-centered, experiential, or psychodynamic therapy would be a challenge to work with this population. For instance, I heard that there was a really good psychotherapy group for the women; a group they really enjoyed. Portuguese immigrants do not usually like attending therapy sessions so I wanted to talk to the therapist who was obviously doing excellent work. She said, sheepishly, it started out as a psychodynamic group but no one would talk about their feelings so now participants bring their knitting and talk about their aches and pains, mirroring the knitting circles in the Azorean Islands.

Despite the increasing acceptance of spirituality and religion in psychology by many psychologists and the APA, therapists are still not routinely trained to competently address religion in therapy (Aten and Hernandez, 2004). Just as we acknowledged the prejudices that we discussed in the socio-cultural domain, we also need to examine the preconceptions that we might have regarding the clients’ spiritual beliefs and practices. Therapists can ask themselves “What stands in the way of clients talking about spiritual issues? How can this session be a spiritual experience?” This can be examined on three levels. The first level explores the personal domain of the therapist. Prejudgments regarding clients’ beliefs might arise in the course of therapy, or the difficulty may involve physical or mental circumstances such as being tired or preoccupied. The second level is structural. For example, the time of day that a client is seen can be a hindrance (e.g., is there one client who is always scheduled as the last client of the day?). The third level is theoretical and involves understanding the assumptions about spirituality as it is recognized (or not) in the psychotherapy model that is being used (as discussed earlier). It is helpful to take time before each session to acknowledge these barriers and to try to minimize their effect.

Once the session is in process, one can promote spiritual discourse in a culturally appropriate way (Frame et al., 1999; Garrett and Wilbur, 1999; Shimabukuro et al., 1999). For instance, when the therapist takes a stance of quietness and compassion (Peavy, 2004) as suggested by wisdom-based therapy, the client may feel the space and respect to fully explore spiritual issues.

In Portugal, at Easter, there are many contemplative activities in which the whole family participates. For instance, the men who participate in Ramirez (an Easter vigil) will walk in bare feet all the way from their house to the church where the Easter procession begins, sometimes many, many kilometers. During Easter there are also many processions happening where all off the family members will be in the procession. They all walk with the cross. These activities are very important to the faith and many people fly in from all over the world to attend. The processions allow the family to embody the faith and for this to be witnessed by the community. There are also Easter processions in Toronto and Boston that are also well attended.

When I (James) asked in a therapy session what made my anxious client feel better, she said reading Bible verses so I integrated that into her treatment. If that is something that gives the person strength, there are also other Christian contemplative practices that could be included in therapy, such as practicing gratitude (Stiendl-Rast, 1984), walking the labyrinth, prayer, and Lectio Divino (reading a Bible passage in a contemplative manner).

Lastly, we value the inquisitive stance of not knowing on the part of the therapist. Barsness (2003) explains from a Christian tradition:

As we enter into the unknowable regions of the patient’s internal world, surrender to the terror of not-knowing, we often unexpectedly find ourselves at the abyss with little left but faith. In order to know we must not know, in order to find ourselves we must lose ourselves, in order to experience we must forfeit explanation. It is in this space that the potential for something other than ourselves, the transcendent revelation of God, can be experienced.

The main limitation with this quantitative study is the limited sample size, but this should be viewed in light of the fact that it is hard to recruit this population. With regard to the qualitative research, the most valuable aspect of ethnography is the depth of engagement, which in ways mirrors the therapeutic relationship; however, the findings must be considered in light of the limitations that result from this type of research relationship. Ethnography positions the researcher as part of the community but the effects of being an outsider cannot be known and, as such, the findings must be considered as the re-telling of the participant’s stories and beliefs of what they chose to share. Although it is human desire to generalize, ethnography is highly situated in time and place and as such the findings of ethnography often ask more questions than that it answers. The results cannot be unilaterally generalized to other Azoreans, Portuguese, or to the experience of other ethnic populations. Another limitation that must be considered is the translation of stories and beliefs from Portuguese to English at many different levels. In some ways, this can be considered a strength, as the researcher is forced to attend to the intricacies of language on a more obvious level as they try to understand not only the spoken but also the unspoken messages of a different culture. In short, this ethnography provided an interpretation of cultural knowledge that deepened the understanding of healing within this immigrant group.

The cultural psychotherapeutic approach demonstrates its value with ethnic minority clients by situating the client within the context of their multi-layered social reality. This approach allows the therapist to better understand the client through exploring the various dimensions of their immediate reality by way of dialog. In this process, the therapist portrays his or her own self as part of the process of therapy, thereby encouraging an open-ended, self-reflexive, dialogic turn of mind. This is reminiscent of ethnographic techniques used by anthropologists (Shweder, 1991). Meanwhile, the client gains a better understanding of his or her own self by coming to understand this way of deriving meaning and by articulating a story or narrative which gives coherence to her or his lived experience.

## Conclusion and Future Directions

At the individual level, our findings provide evidence for the reliability and validity of the Agonias Scale for male Portuguese immigrants. As for female Portuguese immigrants, the scale showed a unidimensional factor structure. This factor addresses psychological and physical aspects, which is in line with our previous qualitative research with Portuguese immigrants in that psychological and physical aspects are seen as integrated. Furthermore, the Agonias Scale was positively correlated to measures of depression and anxiety, with small and medium effect sizes as was hypothesized, indicating that these variables are related, yet distinct and indicative of different aspects of distress. This is noteworthy as agonias is typically conceptualized and treated as anxiety or depression by health-care professionals (James and Prilleltensky, 2002). In the study by James et al. (2004), 63% of the female participants reported having experienced agonias, whereas in the present study 52% of the male respondents reported having experienced agonias. This gender difference in reporting the experience of agonias is in line with other culture bound syndromes for which gender differences have been reported, such as ataques de nervios in the Latino cultures (e.g., Guarnaccia et al., 1993; Jackson, 2006). Furthermore, this is in line with the results of a focus group that was conducted by our research team in Portugal with women and men. Specifically, one question asked participants who is more likely to get agonias, and the participants reported that it is more likely for women to report agonias due the fact that – in the words of one of the male focus group participants – “women are more in touch with their own feelings.”

At the family level, there are a number of new ideas that hadn’t emerged previously. Suffering is a family event and often intergenerational. Receiving healing from the curandeiro is also a family event; the curandeiro may have all family members attend the visit. The curandeiro is Gods’ manifestation on earth and that ability is passed down from generation to generation.

Another new paramount insight that will change how we conduct our research, is that agonias is an infinitely open signifier, which means that it has multiple significations or meanings (de Saussure, 1983). Thus, agonias can be a health problem or an acceptable way to talk about family problems because its meaning is relational (de Saussure, 1974).

The Azorean research revealed new insights at the socio-cultural level as well. First, the importance of understanding that there may be language barriers when moving between the different systems in Portugal such as medical, healers, and community members. Thus the importance being tentative when presenting results becomes important.

Another discovery was that curandeiros are well integrated into the community, and many are employed and attend the local parish. Certainly, when you live on a small remote island the lives of all of the inhabitants are intertwined.

You see how interrelated the levels are and how a discovery at one level can be used to inform future research in a way that we never would have discovered by researching one level. For example, the speculation that Agonias is an open signifier came from the family level but will affect the research that we do at all of the levels. For example we will now give the Agonias Questionnaire (James et al., 2004) several times and see how it changes over time. It would be helpful to give the Hassles Scale (Delongis et al., 1982) at the same time.

In addition, because of the importance of the family level, we should also give the Agonias and Hassles scale to all family members. Accordingly we need to change how we do the qualitative data collection as well. Presently, we interview individuals or members of a focus group when we conduct individual interviews Instead it would be beneficial to interview multiple members of a family together. With parallel studies conducted both in the Azores and in Canada, the similarities and differences in family dynamics could be better understood. This would also yield valuable information regarding how families change with acculturation and exposure to a new majority culture. Another area of the family domain that remains unexplored is how families change over time in the Azores. This has been examined in Canada, and while change is largely attributed to acculturation, there may be other aspects at play. It would be beneficial to interview multiple generational levels of families in the Azores and learn how different generations observe customs and traditions, how they adopt new customs and traditions, including religion, and what conflicts or resolutions emerge.

At the socio-cultural level, we will interview curandeiros to understand their theory of change and treatment rationale. How does language impact the efficacy of their practices? How do they engage in Portuguese communities? How do they conceptualize suffering and healing? How did they become curandeiros and how do they view their roles in the community? Do they collaborate with other mainstream or alternative healers?

At the religio-moral level, unanswered questions that would be worth further investigation include, for both Portuguese Canadians and Portuguese in the Azores, the following:

How do they view traditional healers in the context of their religious beliefs? Are there similarities and differences between the two cultures? How did those patterns come about over time? How do peoples’ religious and other beliefs help them make meaning of their suffering and healing? How do community members integrate mainstream and traditional healing, and how do curandeiros collaborate with other mainstream or alternative healers?

This research on agonias illustrates the importance of using a mixed-method and multi-leveled approach to examine idioms of distress in a culturally sensitive way. Quantitative and qualitative research will be conducted to explore the aforementioned questions.

Traditionally, attempts to understand culture tend to focus on race. For example, Sue and Sue (2007) have historically divided their crucial multicultural counseling texts into race-specific chapters, although recent editions have included more dimensions (i.e., spirituality) and nuances. With Portuguese Canadians, a focus on race by researchers and clinicians may lead to an unfortunate assumption that members of this group are not culturally unique. This could lead to misunderstandings, premature termination of treatment, and unintentional discrimination. As discussed thus far, Portuguese Canadians have rich cultural distinctions, which permeate all aspects of life and health and behavior. They also do experience discrimination and limited economic opportunities in mainstream culture. For instance, while for most immigrants in North America the socio-economic status improves from generation to generation no matter what their race, for the Portuguese, their poverty often does not improve (Reeve, 1998).

It is laudable that Sue and Sue (2007) have included information about culture bound disorders with case examples. The title of that chapter, however, is “Non-Western and Indigenous Healing.” Our concern is that if a therapist had a Portuguese client he or she might not consult that section of the text even though it could be helpful.

We have provided examples from our program of research with the Portuguese both in Canada and the Azorean Islands of Portugal and we have outlined a model for conducting psychotherapy with people of diverse cultural backgrounds based in the theories of clinical and cultural psychology. By integrating psychology and anthropology we gain a more complex understanding of both culture and the individual within his or her cultural context. Our program of research with the Portuguese cultural group both in Canada and Portugal has informed our clinical work with this group and other diverse groups. The model demonstrates its value with ethnic minority clients by situating the clients within the context of their multi-layered social reality. By better understanding how Portuguese suffer and heal, we have sought to develop a model for conducting psychotherapy specific to Portuguese immigrants. We believe the model can be utilized with other non-dominant cultures living in North America.

Table 2 outlines a set of questions that are informed by ethnographic research methods. Our model focuses on understanding the person in a multicontextual way and utilizes the four contexts or domains discussed above: individual, familial, socio-cultural, and religio-moral (see Figure 1). By exploring each level we understand the person in a multicontextual way with the understanding that all levels are interrelated and significant.

**Table 2:** **A model for doing therapy/research with people from other cultures**.

Levels that can be addressed	Questions that can be asked
Individual	What are the symptoms? What are the cures? Are their culture specific disorders in that culture of origin? Who in the community suffer from culture specific disorders (e.g., first generation, second generation, particular locations of country of origin)? Is there overlap with the DSM-IV-TR? Are people being misdiagnosed?
Family	What is your family’s story? What family values are important to each family member? Do children speak language of origin? Do family members acculturate to home country at different rates? How do all members of the family understand the cultures specific disorders? Do children marry people of their culture and how is that viewed by others in the family? Do children maintain the faith traditions of their family of origin? If not how is that?
Socio-cultural	What is the cultural history of the informants? What are the philosophical traditions of the culture of origin? What is the historical context of informants? What is the socio-political context of the informants? What is the socio-economic status of informants? How does SES change from generation to generation?
Religio-moral	What is a good person/family/society? Are their ethical issues that arise? What are the faith/spiritual traditions of their culture of origin? What is the causal ontology for suffering? Are traditional healers in the culture of origin? What brings joy and peace?

## Conflict of Interest Statement

The authors declare that the research was conducted in the absence of any commercial or financial relationships that could be construed as a potential conflict of interest.
